# Dynamic Structure Formation of Peripheral Membrane Proteins

**DOI:** 10.1371/journal.pcbi.1002067

**Published:** 2011-06-23

**Authors:** Diana Morozova, Gernot Guigas, Matthias Weiss

**Affiliations:** 1 Cellular Biophysics Group, German Cancer Research Center, Heidelberg, Germany; 2 Experimental Physics I, University of Bayreuth, Bayreuth, Germany; University of Illinois, United States of America

## Abstract

Using coarse-grained membrane simulations we show here that peripheral membrane proteins can form a multitude of higher-order structures due to membrane-mediated interactions. Peripheral membrane proteins characteristically perturb the lipid bilayer in their vicinity which supports the formation of protein assemblies not only within the same but surprisingly also across opposing leaflets of a bilayer. In addition, we also observed the formation of lipid-protein domains on heteregeneous membranes. The clustering ability of proteins, as quantified via the potential of mean force, is enhanced when radius and hydrophobic penetration depth of the proteins increases. Based on our data, we propose that membrane-mediated cluster formation of peripheral proteins supports protein assembly *in vivo* and hence may play a pivotal role in the formation of templates for signaling cascades and in the emergence of transport intermediates in the secretory pathway.

## Author Summary

Eukaryotic cells are subdivided into a variety of compartments by membranes, i.e. by lipid bilayers into which a multitude of proteins are embedded. About 30% of all protein species in a cell are associated with membranes to perform vital functions, e.g. in signaling and transport pathways. A plethora of membrane-associated proteins, so-called peripheral membrane proteins, penetrate only one monolayer whereas transmembrane proteins span the entire thickness of a lipid bilayer. Despite the crucial role of peripheral membrane proteins in many cellular processes, little is known about the general principles of how they can form higher-order structures that eventually are biologically active units. Using a simulation approach we show and quantify here the influence of membrane-mediated interactions between peripheral membrane proteins. These interactions most likely play a pivotal role in forming higher-order structures in living cells. In particular, template formation in signaling cascades and coat assembly during the formation of transport intermediates may strongly benefit from the phenomena described here.

## Introduction

In recent years, our view on the organization of biomembranes has dramatically changed. The perception has shifted from the fluid mosaic paradigm with virtually no internal organisation [1] to a more inhomogeneous model in which biomembranes are more mosaic than fluid [2]. It is well accepted by now that membranes are not simple homogeneous two-dimensional fluids in which lipids and proteins are randomly dispersed. Rather, membranes are subdivided into (dynamic) domains that are composed of distinct lipids and proteins. In particular, the formulation of the ‘raft hypothesis’ by Simons and colleagues [3] has triggered numerous studies on the existence, composition, and dynamics of membrane microdomains. While the existence of lipid domains in artificial bilayers, i.e. regions with a distinct lipid composition, is by now well established [4], the existence of protein-lipid domains *in vivo* is much less clear and still a matter of debate. It is commonly appreciated, however, that the formation of (transient) higher-order structures on cellular membranes, e.g. during the formation of transport intermediates [5], [6] or in the context of signaling [7], [8], equips biomembranes with a distinct and dynamic substructure.

While traditional biochemical approaches emphasize specific binding events of proteins to and within membranes via cognate motifs, work on membrane domains has also highlighted the role of lipids as mediators of (attractive) interactions. Theoretical and experimental studies have revealed, for example, that a mismatch between the hydrophobic thickness of a lipid bilayer and the length of the hydrophobic transmembrane domain of proteins can support oligomerization and protein sorting [9]–[14]. Indeed, protein assembly due to membrane-mediated interactions was predicted as early as 1984 by Mouritsen and Bloom [15]. Supporting experimental or simulation data, however, had remained ambiguous for a long time. Subsequently, attractive forces between transmembrane proteins due to elastic distortions of the lipid bilayer have been investigated in more detail with continuum models [16]–[18]. But also capillary forces [19], wetting effects [20], curvature [21], [22], membrane fluctuations [23]–[25], and lipid packing [26] have been implicated as a source for membrane-mediated attraction between transmembrane proteins.

A plethora of membrane proteins, however, does not possess hydrophobic transmembrane domains but is only associated with one leaflet of the lipid bilayer. These are typically referred to as ‘integral monotopic proteins’ or ‘peripheral membrane proteins’ (PMPs). Peripheral membrane proteins often contribute to vital cellular functions, e.g. in a multitude of important signaling cascades [27] or during the formation of transport intermediates in the early secretory pathway [28]. Moreover, PMPs frequently form higher-order structures and templates, sometimes even across opposing leaflets of a lipid bilayer. Given that membrane-mediated attraction fosters the assembly of transmembrane proteins, it is tempting to assume that also PMPs benefit from such a generic mechanism. As little theoretical and experimental data on this aspect are available, simulations lend themselves as a powerful tool to elucidate this question.

Inspecting prominent examples of PMPs, e.g. prostaglandin H2 synthase, fatty acid amide hydrolase, but also members of the Ras family, Ras-like GTPases, and Wnt morphogens, it becomes clear that the size of the hydrophobic anchor of PMPs varies considerably (lengths from 3Å to 3 nm, surface area of up to 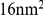). In many cases, its shape can be well approximated by an ellipsoid. Moreover, a recent simulation study showed that the hydrophobic moeity is buried in lipid bilayers to different extents which causes more or less severe deformations of the membrane [29]. It is hence tempting to model PMPs on a coarse-grained level to highlight generic geometrical effects that are not due to the presence of particular lipid species and/or amino acids.

Here, we have used coarse-grained membrane simulations to study several aspects of the dynamics of peripheral membrane proteins. As compared to Molecular Dynamics (MD) approaches, the use of dissipative particle dynamics (DPD) allowed us to explore larger length and time scales for a variety of generic settings. The gain in computational efficiency of our approach comes at the expense of neglecting atomic details (and electrostatics) whereas hydrophobic and hydrodynamic interactions are preserved. Hence, geometric interactions that are due to the latter interactions, are highlighted in our approach.

First, we have studied how PMPs with hydrophobic anchors of varying length and diameter perturb the host membrane locally. Second, we have determined the proteins' diffusion coefficients and relate these results to the well-known Saffman-Delbrück relation. Third, we have examined the tendency of PMPs to form clusters due to non-specific membrane-mediated interactions. As a result, we observed that different types of clusters form when PMPs are residing in the same and in opposite leaflets of a membrane. To explore the stability of these clusters, we have determined the potential of mean force, i.e. the attractive potential, for pairs of PMPs in the same and opposing leaflets of a bilayer. Based on our findings we discuss the relation between local membrane perturbations and the cluster formation ability of PMPs, and we also point out implications of our results on biological processes.

## Results

To elucidate the dynamics of peripheral membrane proteins, we have used dissipative particle dynamics (DPD), a coarse-grained simulation technique that is commonly used to simulate membranes on the mesoscopic scale [30]. In this approach, groups of atoms are combined to effective beads that are the building blocks of more complex constructs, e.g. lipids and proteins. Beads interact via effective potentials and are subject to a thermostat (see Methods for details).

**Figure 1:**
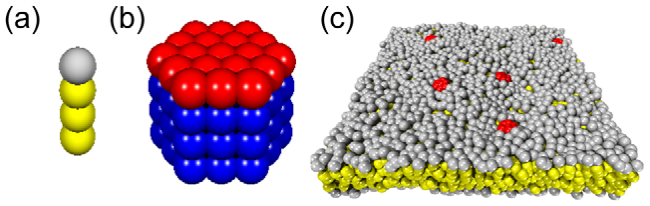
Simulation setup. (a) Model of a L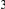 lipid with a single hydrophilic head (grey) and three hydrophobic tail beads (yellow). (b) A model protein 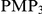 with radius 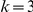 consisted of a single hexagonal layer (diameter 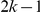 beads) of hydrophilic beads (red) and three layers of hydrophobic tail beads (blue). (c) Snapshot of a membrane of L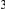 lipids and five embedded copies of 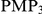 (water not shown for better visibility).

To model a system of water, lipids, and peripheral membrane proteins, we have used three bead types which represent hydrophilic groups, hydrophobic groups, and water. Lipids (denoted as 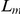) were constructed as linear polymers consisting of a single hydrophilic head group and 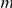 hydrophobic tail beads (Figure 1 a). As a standard lipid we used 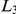. Peripheral membrane proteins (denoted as 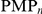) were modeled as cylinders with hexagonal cross section, consisting of one hydrophilic and 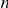 hydrophobic bead layers that form the membrane anchor (Figure 1 b). Beads inside the cylinder were connected with harmonic springs to obtain fairly rigid, compact protein structures similar to those seen in nature as a consequence of covalent and non-covalent interactions of the poly-peptide chain. The proteins' radius 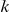 was determined by the number of beads along the hexagonal cross section of a protein, 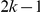. We have concentrated on PMPs with membrane anchor length 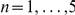. In all simulations, proteins were inserted into pre-assembled lipid bilayers with a patch size of 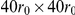 (Figure 1 c). Here, the intrinsic DPD length scale corresponds to 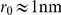. Before recording the positions of all beads as a function of time, systems were equilibrated with a barostat to a tension-free state (cf. Methods).

### Perturbation of lipid bilayers by peripheral membrane proteins

**Figure 2:**
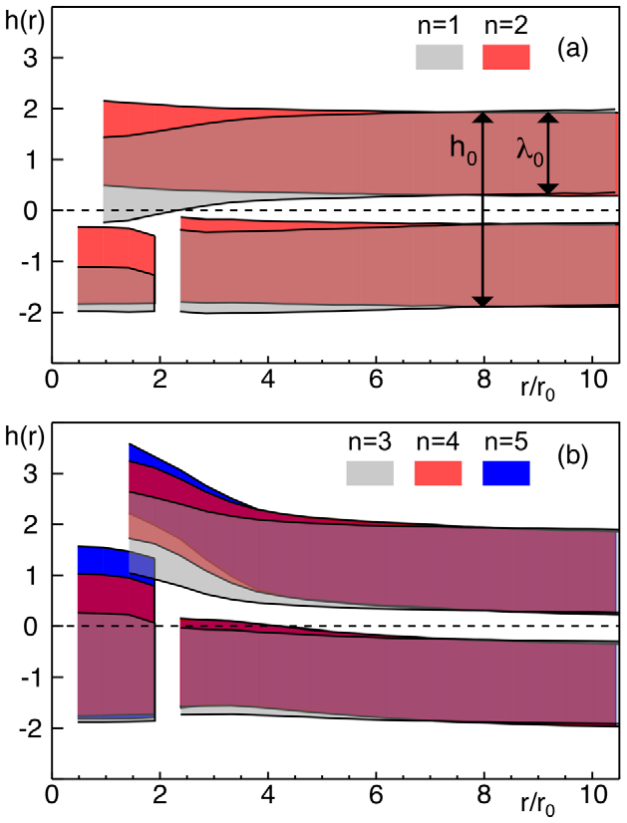
Profile of leaflets in the lipid bilayer next to a protein. When embedding a PMP into a lipid bilayer, the two leaflets are perturbed in a characteristic manner. Regions between the average positions of lipid head and terminal tail beads in the two leaflets are shown as colored stripes. The unperturbed, neutral midplane is indicated as dashed line. A single PMP was inserted in the lower leaflet (shape indicated in region 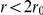). Unperturbed membrane and leaflet thickness, 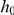 and 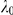, are indicated far away from the protein. (a) When inserting short proteins (i.e. 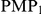 and 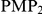) the upper leaflet bended towards the unperturbed midplane while the lower leaflet remained almost unperturbed. (b) For longer membrane anchors (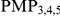), the upper leaflet bended away from the midplane due to the steric interference of the lipids with the opposing hydrophobic moiety of the protein. The lower leaflet bended only slightly inwards, hence resulting in a local thickening of the membrane. Also, the thickness of the upper leaflet, 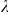, was slightly reduced due to steric compression.

We first characterized a lipid bilayer consisting of 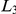 lipids without any embedded proteins. The thickness of this bilayer was 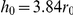 (average distance between lipid head group centers of opposing leaflets; cf. Figure 2). The thickness of one leaflet was 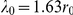 (average distance between head group centers and terminal tail bead centers within a leaflet; cf. Figure 2). The distance between the leaflets was 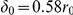 (average distance between the centers of terminal tail beads in opposing leaflets; cf. Figure 2). The orientational order parameter of the lipids, 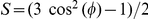, with 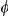 being the average angle between lipids and the bilayer normal, assumed a value 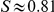. Given the extremes (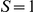 when lipids are oriented parallel to the bilayer normal, 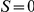 for random orientation), the observed value indicates a fairly ordered yet fluid lipid bilayer.

In order to probe alterations of the bilayer's shape and the lipids' configuration upon embedding a peripheral membrane protein into the membrane, we inserted a single 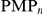 with radius 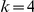 and increasing hydrophobic length (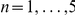) into the bilayer. After equilibration, the tilting of the protein with respect to the bilayer normal was very modest (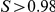). Only the shortest protein, 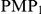 , showed a somewhat enhanced tilting (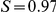) with enhanced fluctuations. The latter is also reflected in the distribution of protein tilting angles that was slightly broader for 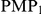 than for all other PMPs (data not shown). This observation suggests that 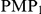 assumes a slightly less stable configuration within the membrane as compared to proteins with longer hydrophobic moieties.

Next, we monitored the membrane's perturbed cross-section profile, i.e. the average positions of lipid head group centers and terminal lipid tail bead centers. In general, marked perturbations were visible in membrane regions close to the protein (Figure 2 a). When the protein's hydrophobic moiety did not reach into the opposing leaflet (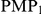 and 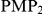 , Figure 2 a), the opposing leaflet was partially bent towards the unperturbed midplane. The leaflet in which the protein was embedded remained almost unperturbed and hence the membrane was thinner near to the protein, i.e. 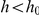. The least perturbation was seen for 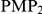 for which the length of the hydrophobic moiety (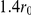) matched approximately the thickness of the unperturbed leaflet (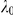). For PMPs with a longer hydrophobic moiety (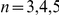) the opposing leaflet bended away from the midplane (Figure 2 b). The absolute deflections from the midplane at the boundary of the PMP grew almost linearly with the length of the hydrophobic moiety, i.e. 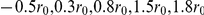 for 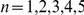. The leaflet in which the PMP was embedded only bended slightly into the same direction (Figure 2 b), i.e. a net increase in membrane thickness (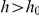) near to the protein emerged.

**Figure 3:**
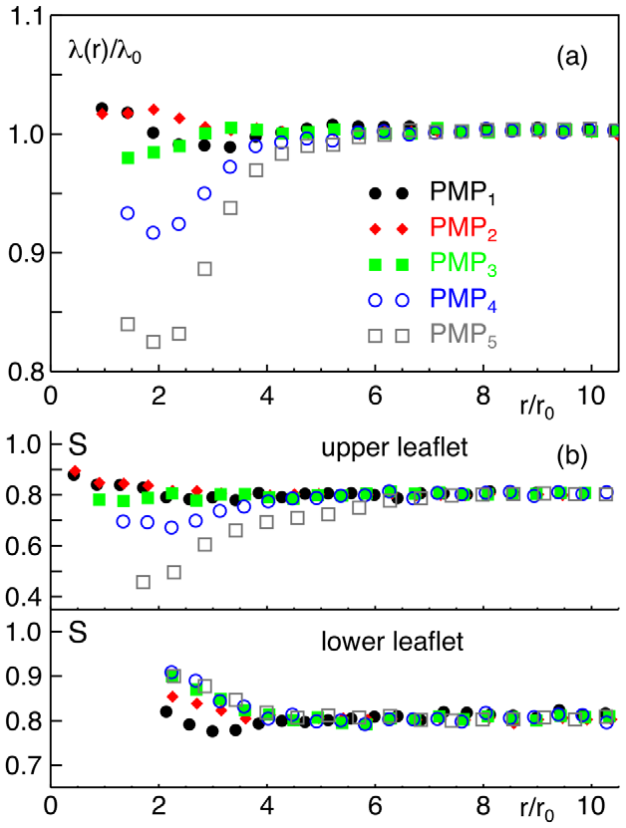
Membrane perturbations when inserting a peripheral membrane protein. (a) When inserting a 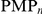 construct, the thickness 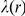 of the opposing leaflet is significantly altered near to the protein with respect to the unperturbed value 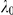. The compression of the leaflet increases when the length 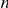 of the hydrophobic moiety is increased. (b) The lipids' orientational order parameter 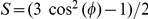 (with 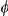 being the lipids' average angle to the bilayer normal) in the upper and lower leaflet is also affected in the vicinity of a PMP. Only far away from the protein a convergence towards the unperturbed value is observed. In agreement with the local compression of the upper leaflet, a stronger tilting of lipids is observed directly opposite to the PMP. Legend as in (a).

We next determined the thickness of the membrane leaflets, 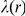, i.e. the average distance of lipid heads and tail ends within a leaflet. The leaflet in which the PMP was embedded changed its thickness only marginally whatever protein was inserted (data not shown). The leaflet opposite to the PMP, however, showed significant changes depending on the length of the hydrophobic moiety (Figure 3 a). In particular, for 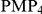 and 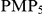 a strong compression of the leaflet emerged while a length 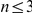 of the hydrophobic moiety only had a negligible effect since the hydrophobic moiety was too short to penetrate strongly into the opposing leaflet. This finding for 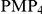 and 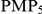 is corroborated by the observation that lipids showed a decrease in the orientational parameter 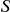, i.e. they aligned less with the bilayer normal, when being situated right opposite to these proteins (Figure 3 b). However, also shorter proteins induced a change of lipid orientation in the opposing leaflet: Here, the lipids aligned more strongly with the bilayer normal, i.e. 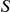 increased. Within the leaflet in which the protein was embedded, the lipid orientation was also affected near to the PMP. Near to 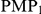 , lipids were tilted more strongly, whereas they rather aligned better along the surface normal for proteins with a longer hydrophobic moiety. Thus, in all cases the lipids' freedom is constrained, i.e. 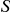 deviates from the unperturbed value, which is entropically unfavorable.

To complement the above results, we also analyzed the average distance between the leaflets, i.e. the average distance of the centers of the terminal lipid beads in each leaflet (cf. also Figure 2). As a result, we found a reduced distance (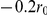) near to the shortest protein, 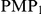 , while for 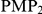 no change with respect to 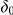 was observed. For longer proteins (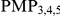) the distance increased by 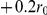, 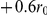, and 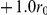, respectively.

In summary, our results on lipid tilting and bilayer thickness are in agreement with previous observations [31]. Going beyond these results, we also have quantified perturbations of each monolayer and the altered coupling length of the monolayers.

### Diffusion of peripheral membrane proteins

Having quantified the local membrane perturbations induced by embedding a peripheral membrane protein into a lipid bilayer, we next asked how these proteins diffuse within the membrane. For transmembrane proteins the famous Saffman-Delbruck relation [32] predicts a logarithmic size-dependence for small radii (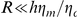)
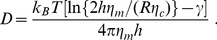
(1)Here, 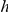 and 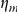 are the thickness and viscosity of the membrane, 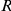 is the protein radius, 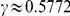 is Euler's constant, and 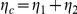 is the sum of the viscosities of the fluid above (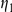) and below (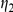) the membrane [33]. The validity of Eq. (1) has been confirmed by simulations [34] and experiments [35]–[37].

To probe whether the size-dependent diffusion of peripheral membrane proteins also is described by Eq. (1) , we embedded single PMPs (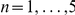) of varying radii (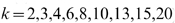) into a lipid bilayer (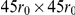). Owing to the use of soft-core potentials in DPD, momentum and mass transport happen on the same time scale, hence allowing for a sound quantification of transport properties even in fairly small systems. After equilibration, a protein's position was tracked for 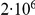 steps. During this period, proteins moved on average a distance of 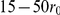. From the time series of positions, we determined the protein's (time-averaged) mean square displacement 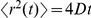. The diffusion coefficient 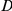 was obtained subsequently by fitting the mean square displacement. The uncertainty in determining the diffusion coeficient was less than 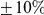 as judged from several runs for the same parameter settings. As a reference we also determined the diffusion coefficients of a transmembrane protein with 5 hydrophobic bead layers and of a single lipid. The latter yielded the diffusion coefficient 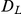 to which all diffusion coefficients of proteins were compared.

Similarly to the case of a membrane that separates two fluids of different viscosities, we anicipated 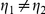 for PMPs since a protein's top feels the surrounding water while its bottom is burried in the core of the bilayer. Therefore, 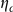 was expected to vary with the penetration depth 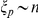 of the protein. Consequently, 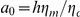 was used as an open fit parameter in Eq. (1) . The second fit parameter was 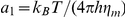.

**Figure 4:**
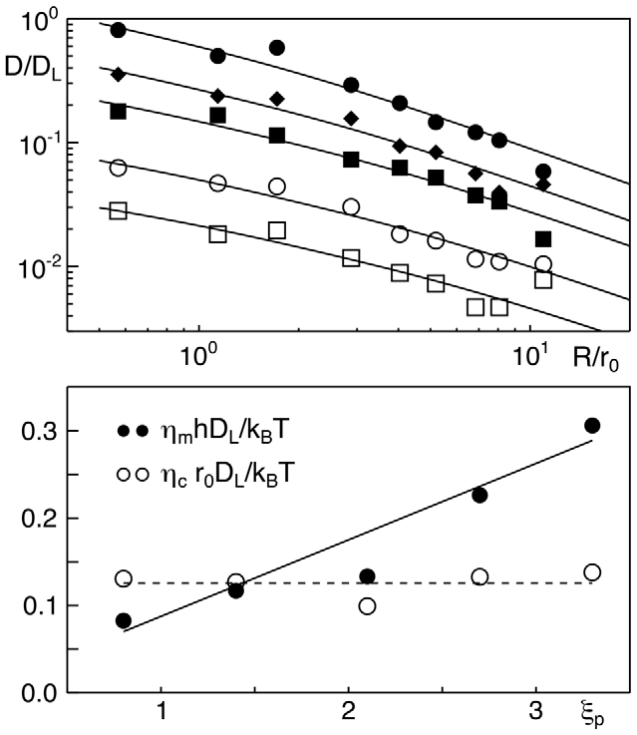
Size-dependent diffusion coefficients of peripheral membrane proteins. (a) The dependence of a peripheral membrane protein's diffusion coefficient 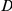 on its radius 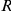 is well described by Eq. (1) ; 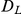 denotes the diffusion coefficient of a lipid, 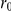 is the size of a simulation bead. Proteins with 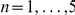 are denoted by filled circles, diamonds, squares, and open circles, squares, respectively. Error bars are smaller than symbol size. Please note: For better visibility, curves corresponding to 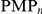 have been shifted by a factor 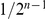; unshifted curves approximately coincide with the uppermost curve. (b) The viscosity 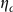 in Eq. (1) (shown here as dimensionless quantity 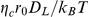) does not vary systematically when increasing the penetration depth 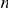 of a protein. In contrast, the surface viscosity 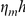 (shown as dimensionless quantity 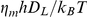) shows a linear increase with the penetration depth 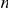.

As result, we found that Eq. (1) provides an excellent fit to the size-dependent diffusion coefficients of all simulated proteins (Figure 4 a). In contrast to our expectation, 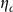 did not show a significant variation when the protein's penetration depth 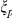 was increased by extending the length 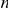 of the hydrophobic moiety (Figure 4 b). However, the change of 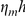 was proportional to the length 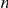 of the protein's hydrophobic moiety, 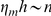 (Figure 4 b). This result reflects that the protein does not experience the full viscous drag (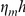) of a transmembrane protein but only a smaller portion due to the smaller penetration depth. Notably, the diffusion coefficient for 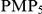 almost coincided with that of a transmembrane protein. In our simulations we did not observe a significant annulus of lipids traveling with the protein. Most likely, coarse-graining and the use of soft potentials (i.e. a low ratio of momentum to mass propagation inherent to DPD) softens the emergence of these anticipated short-ranged, dynamics assemblies.

When dissecting 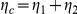, i.e. extracting the membrane's contribution, it is noteworthy that 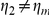 albeit both arise from the friction with lipids. We attribute this difference to the different contacts that a PMP makes with lipids: While the protein's bottom is mainly in contact with lipid tail beads, the lateral face is immersed within elongated lipid chains. Comparing the diffusion coefficients of PMPs, we find that they vary in maximum about 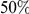 between the lowest (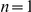) and the largest penetration depth (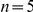). Hence, similarly to transmembrane proteins with a hydrophobic mismatch [38], [39] the variation of the diffusive mobility is very moderate.

### Clustering of peripheral membrane proteins within the same leaflet

Given that peripheral membrane proteins perturb the lipid bilayer and reduce the lipids' degrees of freedom, one may expect a dynamic, entropy-driven clustering of proteins in analogy to observations made for transmembrane proteins [10]. We therefore studied as a first step the clustering behavior of two peripheral membrane proteins that reside in the same leaflet.

To probe and characterize membrane-mediated interactions of peripheral membrane proteins, we determined the free energy of association between two PMPs. To this end, we quantified the potential of mean force (PMF), 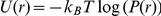, via an umbrella sampling of the inter-protein distance distribution 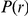 (see [40], [41] for a detailed introduction). Here, we have restricted our analysis to pairs of identical proteins and varied the proteins' radii (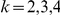) and lengths of the hydrophobic moiety (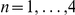).

**Figure 5:**
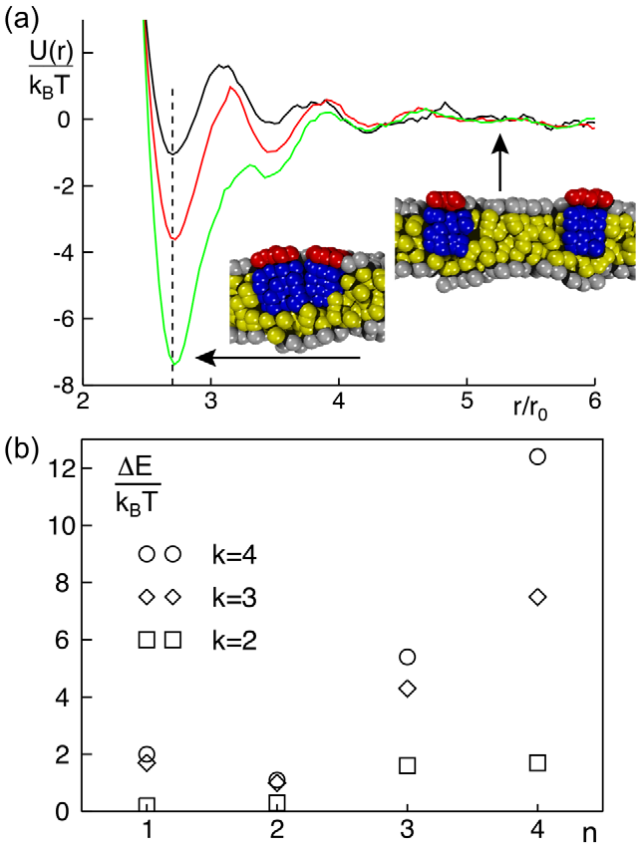
Dimerization of peripheral membrane proteins. (a) Representative potentials of mean force, 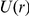, of two PMPs (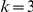) residing in the same leaflet. The minimum of 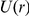 is located at the anticipated distance where proteins touch each other (dashed line). The binding energy 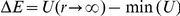 increases with growing hydrophobic length of the protein (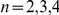, shown in black, red and green, respectively). Representative snapshots indicate the protein configuration in the bound and unbound state. Hydrophilic and hydrophobic groups are shown in red/grey and blue/yellow, respectively. (b) The binding energy 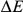 increases with the length of the hydrophobic moiety, 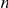. While one observes for small radii (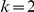) only a very small increase in the dimerization energy, a strong increase is seen for larger radii (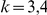).

As a result, we found that the PMF for all PMP pairs within the same leaflet had a deep minimum at small inter-protein distances (Figure 5 a), i.e. when the proteins are located side-by-side. This feature indicates a bound state, i.e. the formation of a (transient) dimer. Beyond the minimum in the PMF, 2–3 weak side minima emerged which most likely reflect metastable configurations with 1–2 lipids in between the PMPs. For large inter-protein distances the PMF converges to a constant, i.e. PMPs do not interact over larger distances.

The depth of the main minimum as compared to the PMF value far away from the protein, i.e. 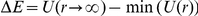, reflects the strength of binding and hence determines the dimer stability. Depending on the length of the hydrophobic moiety and on the protein radius, we observed values in the range 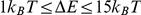 (Figure 5 b). Comparable experimental values were reported for membrane-mediated association of transmembrane helices (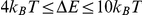) [42]. We observed the weakest binding energy 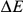 for 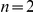 and the strongest binding for 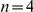 (Figure 5 b). For a given length of the hydrophobic moiety, 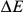 increased with the protein radii 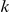. Interestingly, the width of the main minimum hardly changed with protein radius when PMPs were fairly short (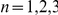: width 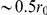). For longer hydrophobic moieties (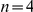), the width increased stepwise with the radius (up to 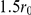).

To relate the PMF to the actual stability of dimers, we next calculated the mean first passage times 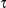 from the energy minimum to the unbound state. Solving the Kramers problem for a square potential well, the prediction is that 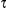 depends quadratically on the potential width and exponentially on the potential depth (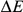). Hence, the dominant contribution for the dimer stability is 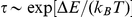. As a result of the full integration, we found 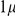
s
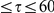
ms (cf. Tables S1,S2). For comparison, we also monitored the lifetime of pre-formed dimers that may dissociate due to diffusive motion. The accessible lifetimes of this approach agreed very well with the mean first passage times calculated above.

In summary, we have found a membrane-mediated attraction between PMPs in the same leaflet that is enhanced for increasing length and radius of the hydrophobic moiety. In particular, we observed that potential depths were rather small (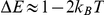) when the proteins' hydrophobic anchor did not penetrate the opposite leaflet. For proteins with hydrophobic lengths 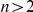 and radii 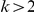, we found larger values of 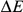 that highlight a significant tendency for proteins to dimerize. It is worth noting here that clustering of PMPs within the same leaflet was also investigated in a related simulation study [31], yet the PMF was not determined. The authors observed an increase of cluster sizes when the proteins penetrated also the opposing leaflet whereas almost no clustering was observed for proteins that were restricted to a single leaflet. These observations are consistent with the energy values found here.

### Clustering of peripheral membrane proteins in opposite leaflets

To elucidate whether peripheral membrane proteins do also interact and potentially dimerize when being situated in opposing leaflets of a bilayer, we inserted a single protein in each of the two leaflets (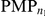 and 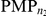) and determined again the potential of mean force (PMF). As above, we systematically varied the protein radii (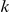) and length of the hydrophobic moieties (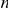).

**Figure 6:**
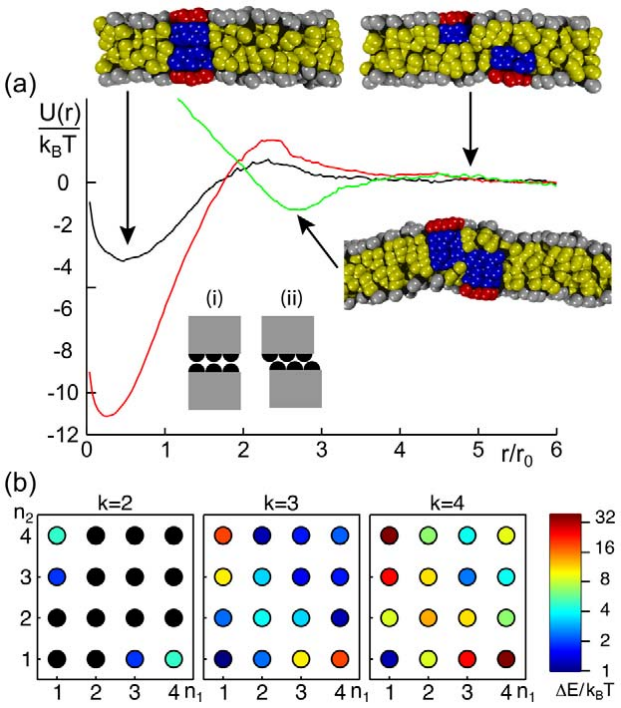
Dimerization of two peripheral membrane proteins in opposing leaflets. (a) Representative potentials of mean force, 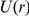, of two PMPs (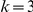) residing in opposing leaflets. For combinations of sufficiently short proteins (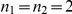, black curve; 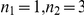, red curve) the minimum of 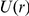 emerges at vanishing distances. For 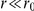 a slight increase of 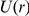 is observed as a result of the PMPs' construction via finite beads, i.e. configuration (i) is energetically less favorable than arrangement (ii). For longer proteins (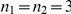, green curve), a side-by-side arrangement of PMPs is observed, and the minimum of 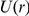 hence is shifted to larger distances. Representative snapshots indicate the discussed arrangements; hydrophilic and hydrophobic groups are shown in red/grey and blue/yellow, respectively. (b) Phase diagram for the dimerization ability when changing the membrane anchor lengths 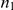 and 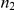 of a pair of PMPs. Color-coded values of 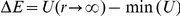 indicate the binding strength. Please note the logarithmic scale of the color-coding; values with 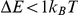 are marked in black.

Similar to our results above, all PMFs showed a deep minimum at small inter-protein distance which indicated a metastable dimerized state (Figure 6 a). In contrast to PMPs in the same leaflet, the minimum of the PMF was here typically situated at very small distance (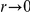), i.e. PMPs formed cross-leaflet dimers in which the individual proteins assumed a stacked configuration (cf. snapshots in Figure 6 a). The global minimum was sometimes followed by a small repulsive barrier at intermediate distances which has to be overcome to reach the dimerized state. The binding energy, 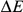, depended on the protein radius 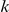 and the combination of hydrophobic lengths 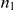, 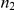 (Figure 6 b). The strongest binding was found for combinations of a very short and a long PMP (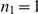 and 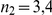). Combinations that involved a PMP that matched best into a single monolayer (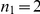) showed medium values of 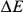 (with 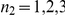). The weakest binding was seen for 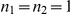, 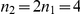, and combinations of 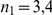 and 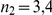. For increasing protein radii, the characteristic shape of the PMF was preserved, yet the depth of the potential well (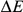) increased, i.e. dimers became more stable. It is worth noting here that an extraordinary strong binding, 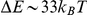, emerged for the largest radius (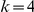, i.e. a protein diameter of 3–4 nm). Given the simplicity of our model and the unavoidable finite-size effects that may suppress part of the relevant undulations and peristaltic bilayer modes, this value may be an overestimation. Despite some numerical corrections that one may anticipate for these extreme cases, the overall tendency to stabilize dimers for increasing radii is a consistent result of our simulations.

The shape of the dimer depended significantly on the length of the hydrophobic moietes of the involved PMPs (cf. snapshots in Figure 6 a). When the hydrophobic length of the two PMPs was small enough (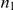 or 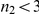), proteins formed a stack-like dimer (bottom to bottom arrangement), and the width of the global minimum in the PMF was roughly the diameter of the proteins. Hence, proteins attracted each other as long as they overlapped. For larger distances, a small repulsive part emerged in the PMF, reflecting the work that has to be done to rearrange lipids and proteins before the dimer can form. The height of this repulsive barrier was largest when both proteins were very short (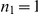, 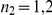) or, to a lesser extent, when one was very long (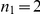, 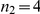). In particular, the combination of two very short proteins (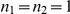) yielded an energy barrier of 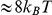 so that a spontaneous dimerization of these proteins is very improbable. When the hydrophobic anchors of both proteins penetrated the opposing leaflet (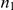 and 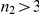), the dimerization partners were located side by side (cf. snapshot in Figure 6 a). The attractive basin of the PMF in this case was very similar to the results for PMPs in the same leaflet, i.e. beyond an inter-protein distance of 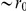 virtually no attraction could be observed.

As before, we also calculated the mean first passage times 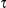 as a measure for the dimer lifetime (cf. Tables S3,S4). As compared to PMPs residing in the same leaflet, cross-leaflet dimers were generally more stable due to deeper and wider minima in the PMF. Escape times ranged from 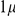
s to 100 ms and beyond, indicating that a broad range of fairly stable cross-leaflet dimers can form. Again, probing the stability of a pre-formed dimer by monitoring the diffusively driven dissociation of the proteins yielded similar lifetimes.

Summarizing our results, we have found that altering the radius and/or the hydrophobic length of a pair of peripheral membrane proteins provides a means to induce cross-leaflet clustering of initially independent proteins. Such a dimerization event may be regarded as the formation of an effective, metastable transmembrane protein. Our observations further suggest that the tendency of two proteins to form such a membrane-spanning dimer depends on two parameters: (a) on the perturbation of the membrane by each individual protein when the protein is either longer or shorter than the thickness of the host monolayer, and (b) on the hydrophobic matching of the effective transmembrane domain of the cross-leaflet dimer. In agreement with this statement, we found that a combination of proteins with 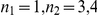 showed a strong dimerization already at the smallest radii (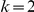). While the length of each of these proteins matches the monolayer thickness only badly, the resulting dimer has only a very small hydrophobic mismatch (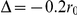 and 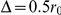, respectively). In contrast, protein combinations 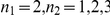 showed longer escape times only for large radii (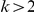), since 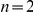 yields the best match to the monolayer thickness. In case of a very strong mismatch of the dimer (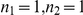; 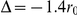) a significant dimerization with long escape times is only seen for very large radii 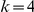.

### Establishing larger protein assemblies

To examine whether a larger number of peripheral membrane proteins in opposing leaflets can show higher-order oligomerization, we inserted 5 PMPs of radius 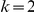 in each leaflet at random positions. Proteins were free to diffuse and to spontaneously aggregate. For larger radii (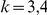), we embedded only 3 proteins in each leaflet to avoid finite size effects. Again, the lengths of the hydrophobic moieties 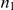, 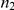 of the PMPs were varied systematically.

**Figure 7:**
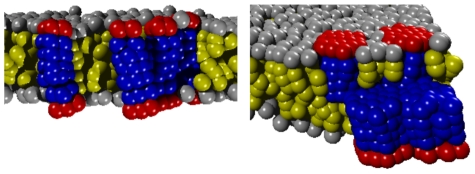
Higher-order clusters of peripheral membrane proteins. Cross-leaflet dimers (cf. Figure 6) showed the ability to form larger oligomers, e.g. trimers of dimers for 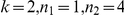 (left) or large side-by-side clusters for 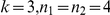 (right).

As a result, we found in the first instance again the spontaneous formation of cross-leaflet dimers of PMPs which we had observed already before (cf. Figure 6). Subsequently, however, these dimers showed the ability to form even larger assemblies when dimers were long-lived enough to meet each other via diffusion (Figure 7 a). In particular, we observed the formation of trimers of pre-assembled dimers for a combination of 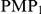 and 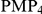 . From the simulation time course, we determined the lifetime of these trimers. They increased with increasing radii from 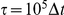 (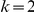), to 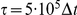 (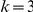), and 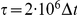 (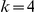).

For a combination of 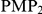 and 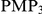 we observed the formation of cross-leaflet dimers for the smallest radius (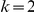) without any subsequent clustering. For larger radii (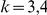), however, again trimers of cross-leaflet dimers with increasing stability emerged. In the described cases, the cross-leaflet dimers had a length of 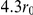 (average distance of head groups in the upper and lower protein). The effective transmembrane part of a cross-leaflet dimer therefore induces a small, assembly-driving hydrophobic mismatch (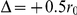) with the unperturbed bilayer.

We also observed a dimerization/trimerization of dimers for large radii (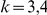) when pre-formed dimers had a negative hydrophobic mismatch, i.e. for 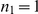 and 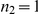 (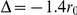), for 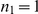 and 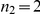 (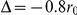), and for 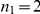 and 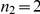 (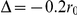). No higher oligomerization of dimers was seen for 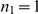 and 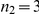, where the preformed dimers had a vanishing mismatch (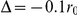). We deduce from these findings that an absolute hydrophobic mismatch 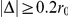 of a (meta)stable dimer of two PMPs yields a membrane-mediated attraction that can cause higher oligomerization of dimers. This outcome is in agreement with the notion that even a small hydrophobic mismatch of a transmembrane protein yields an attractive, membrane-mediated interaction that can drive transient clustering [10], [14]. Cross-leaflet dimers hence act in this respect similarly to transmembrane proteins.

The formation of a variant type of large clusters was observed when the PMPs in different leaflets were penetrating the opposing leaflet (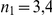, 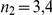) (Figure 7 b). These clusters did not consist of effective transmembrane entities as before but rather were a side-by-side assembly of proteins. The average number of proteins in the clusters was 3–6 and the clusters' lifetimes were 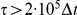 (as determined from the simulation time course). Both, cluster size and stability increased again with an increasing hydrophobic length and radius of the involved PMPs. The strongest clustering was observed for the largest radius (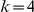). Here, all proteins in the membrane assembled to just one large cluster that was stable for the entire simulation (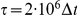).

**Figure 8:**
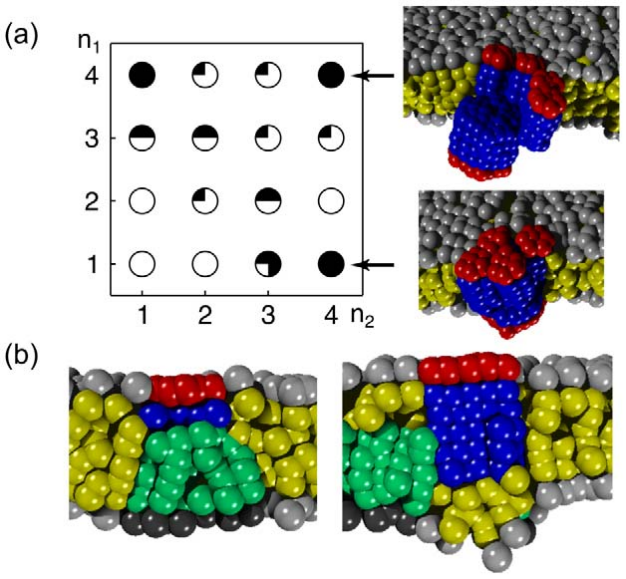
Cross-leaflet clusters of proteins and lipids. (a) Using proteins with different radii, one frequently observes a clustering of small PMPs beneath a large protein in the opposing leaflet. In the lower leaflet a single PMP with radius 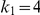 and length 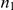 of the hydrophobic moiety was embedded, while the opposing leaflet hosted four PMPs with radius 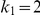 and length 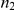 of the hydrophobic moiety. Depending on the length of the hydrophobic moieties up to four small PMPs assembled stably above a single, larger PMP in the opposing leaflet (filling degree of circles encoding the number of small PMPs). Two representative snapshots illustrate representative phenotypes. (b) On inhomogeneous membranes PMPs also frequently assemble beneath or next to a (thick) lipid microdomain in the opposing leaflet.

To complement our results we also performed simulations in which a single protein with a large radius (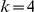) was embedded in the lower membrane leaflet while 5 proteins with small radii (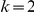) were situated in the upper leaflet. As a result, we observed that the small proteins showed a tendency to assemble above the large protein situated in the opposing leaflet (Figure 8 a). The degree of assembly, i.e. the number of small proteins that stably oligomerized with the large protein, depended on the combination of lengths of the proteins' hydrophobic moieties. A strong oligomerization with lifetimes 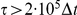 was observed, for example, for 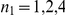 (large protein) and 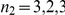 (small proteins). For these cases, typically two small proteins assembled above one large protein. The strongest effect was seen for 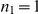 and 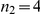: Four small proteins assembled above the large protein and stayed there for the entire simulation time (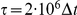). Indeed, for sterical reasons at best four small proteins (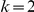) can be placed under one big protein (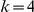). When small and large proteins both were long enough to reach into the opposing leaflet, i.e. 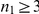 and 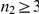, we found that the small proteins did not locate above but rather at the rim of the large protein (cf. Figure 8 a). We found a remarkably large cluster with 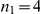, 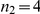, where up to four small proteins surrounded the large protein, hence establishing a pentamer (lifetime 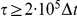); smaller oligomers with the same protein length configuration were stable over the entire simulation (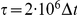).

Finally, we probed whether the above described assembly of proteins in opposing leaflets also occured when one of the proteins was replaced by a lipid microdomain. To this end, we created an asymmetric bilayer consisting of two lipid types, 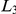 and 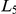, where 10% of the lipids in the upper leaflet were long lipids, 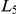. Due to the imposed parameters (cf. Methods) long lipids formed a thick microdomain (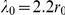, average distance lipid head to lipid tail) in the upper leaflet. A single PMP was then inserted in the opposing, homogenous leaflet. Again, we systematically varied the protein radius (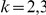) and hydrophobic length (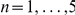). As a result, we observed a co-localization of 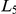 lipids and the PMP with the precise conformation depending on the parameters of the protein. In general, proteins of short and medium length (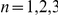) assembled above the lipid microdomain (Figure 8 b). In contrast, for 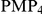 and 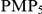 we observed that long lipids preferentially localized to the rim of the protein, sometimes even encircling it. Thus, also clustering of PMPs and (thick) lipid microdomains in the opposing leaflet is a means of structure formation on membranes.

## Discussion

In summary, we have shown that peripheral membrane proteins perturb their lipid environment locally by bending the membrane's leaflets and altering the tilt angle of lipids. These changes depend on the PMP's radius and on the length of its hydrophobic moiety. Determining the potential of mean force (PMF) for protein pairs, we have found a strong attractive interaction for various protein combinations. This membrane-mediated attraction supports the dimerization within one leaflet of the membrane as well as the formation of cross-leaflet dimers. The binding energy 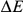 depended strongly on the PMPs' radius and hydrophobic length for both, in-plane and cross-leaflet dimers. The associated mean first passage times, i.e. the dimer lifetime, varied with 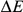 and the width of the PMF minimum. Inserting more than two PMPs into a membrane resulted in the formation of higher-order clusters with variable shapes and substructures. Finally, on inhomogeneous membranes we observed that PMPs frequently associated with (thick) lipid microdomains.

Having observed the formation of dimers and higher-order oligomers without imposing attractive interactions or binding sites, one may wonder what is the driving force behind this structure formation. It is tempting to draw the analogy to transmembrane proteins, for which the minimization of the bilayer perturbations is a driving force for structure formation [10], [17]. Unlike the case of transmembrane proteins, we are not aware of any continuum theory that one could apply easily to the problem studied here. Therefore, we will restrict ourselves to a phenomenological discussion of the problem and relate membrane perturbations, protein geometry and clustering qualitatively.

Continuum mechanics predicts an increase in the free energy when membranes undergo bending and/or thickness compression [16]–[18]. A similar energetic penalty can be anticipated when instead of a bilayer two single leaflets are affected individually by bending and compression/expansion. Confining the configuration space of lipids by altering their tilt angles near to proteins decreases the system's entropy. This may be compensated by clustering proteins, i.e. by minimizing the lipid-protein contact area. As long as the associated gain in entropy from liberating the lipids overcompensates the decrease of the proteins' mixing entropy, cluster formation is supported in analogy to the formation of micelles by amphiphiles in water. In addition, altering the coupling between the bilayer leaflets, i.e. changing the distance between the monolayers, due to inserting a PMP may amplify or weaken bilayer fluctuations. All of the aforementioned contributions are expected to vanish when the protein length matches the thickness of the leaflet (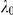). In contrast, with a growing mismatch between the length of the PMP's hydrophobic moiety and the leaflet thickness, the perturbations are expected to grow and clustering hence can become a favorable means to relax stress in the bilayer. Moreover, the membrane area in which lipids are perturbed by the presence of a protein increases with the PMP's radius, which explaines why the protein's tendency to cluster increased with its radius. Notably, PMPs in opposing leaflets dimerized not only when the resulting effective transmembrane dimer matched the thickness of the bilayer. Cross-leaflet clustering (in a side-by-side fashion) was also observed when each of the two proteins penetrated the opposing leaflet.

Comparing our results to other studies, we note that different degrees of coarse graining have been employed to study clustering of transmembrane proteins in the presence of a hydrophobic mismatch [10], [43], [44]. Determining the PMF for these proteins for different hydrophobic mismatches revealed binding energies in the range 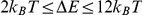 [10], [44]. For PMPs, we have found here a similar range of binding energies, suggesting that in both cases membrane-mediated interactions, e.g. local elastic perturbations of the bilayer, are responsible for the clustering. Moreover, our results on 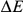 compare favorably to experimental data on the association energies of transmembrane helices (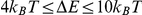) [42]. Clustering can also occur, however, via mechanisms in which such deformations do not play a role. If a protein preferentially adsorbs to one lipid species in a segregating mixture of lipids near the critical point, a lipid-mediated attraction of proteins emerges [45].

To adress the influence of specific sequence motifs in the oligomerizing proteins, a more refined technique like MD would have to be employed. However, coarse-grained MD studies on glycophorin A revealed, for example, that despite the simplification of the chosen model still the main features of a dimerization event and even mutation-induced perturbations were well captured [46]. In coarse-grained MD, typically one or two beads are mapped to a amino acid, and the variety of bead types and interaction potentials is therefore higher than in DPD. Furthermore, such an approach allows for a direct conversion of the structure into an all-atom model while DPD simulations rather deal with a more simplified model on larger length scales. This lack of atomic details in DPD is counterbalanced by the capability to study larger systems over longer periods of time. In particular, geometry- and hydrodynamics-related phenomena are highlighted in DPD simulations. Aiming at generic results that are not restricted to a particular amino acid composition, DPD is therefore a valuable tool to study the collective behavior of proteins in membranes.

Our findings highlight an important, yet often discarded aspect of molecular interactions on biomembranes. When estimating the planar distribution of membrane constituents, typically specific interactions like hydrogen bonds and electrostatics between cognate residues are taken into account. Our study, however, demonstrates that fairly strong non-specific interactions can exist between peripheral membrane proteins simply due to local perturbations of the lipid bilayer: The mere presence of PMPs in a membrane leaflet can lead to spontaneous oligomerization events and hence support the structuring of biomembranes. Not only did we observe protein oligomers but also the formation of lipid-protein clusters. These membrane-mediated attractions potentially represent a significant contribution to the interactions of proteins. They may serve, for example, as a promoter of loose associations from which specific binding events become possible in the first place. Indeed, not only the encounter rate of PMPs is increased by membrane-mediated attractions but also the dwell time in the reaction zone close to each other is enhanced. Both effects support the probability that a specific reaction can take place. Thus, membrane-mediated oligomerization could serve as a preselection or sorting mechanism that facilitates signaling events, enzymatic reactions, or the formation of transport intermediates.

A well-known cross-leaflet dimer, for example, is the channel protein gramicidin A, a dimer of two monomeric units each of which is located in one leaflet of the membrane. These two units localize opposite to each other and form a cross-leaflet dimer similar to the ones we have observed in our simulations. In our model, the protein with 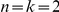, would be an appropriate representative of a gramicidin monomer. Even though gramicidin dimer formation depends on specific interactions of the monomers, our results suggest that unspecific lipid-mediated interactions may play a pivotal role in the early assembly of the channel.

Our findings also imply a simple yet tunable means to transfer information across a membrane. Changing the shape of a PMP, e.g. in the outer membrane leaflet, also perturbs the inner leaflet which may lead to an oligomerization with PMPs in the inner leaflet. As signal propagation at the plasma membranes relies on cross-membrane information transfer, our data supports the hypothesis that transmembrane proteins are not mandatory for signaling cascades at the plasma membrane but that peripheral membrane proteins alone are in principle sufficient. Indeed, experimental evidence for signal transduction on the basis of peripheral membrane proteins has been reported recently (see, e.g., [47]–[49]). Typically, signal propagation is initiated by the binding of an extracellular ligand to a transmembrane receptor in the plasma membrane and a subsequent oligomerization of the receptor. This oligomerization event induces a (de)phosphorylation on the intracellular side of the membrane, hence triggering downstream parts of the pathway. Based on our simulations we put forward the hypothesis that binding of a ligand to a PMP in the plasma membrane's extracellular leaflet may increase the protein's radius and/or the length of its hydrophobic moiety, hence inducing a dimerization with another PMP in the intracellular leaflet. This dimer acts like an effective transmembrane protein and can trigger signaling by oligomerization in very much the same way as actual transmembrane receptors do. Alternatively, a ligand may cross-link several PMPs in the extracellular leaflet which leads to an oligomerization of smaller peripheral proteins in the intracellular leaflet, thus producing a template for triggering downstream signal cascades.

### Conclusion and outlook

In conclusion, we have shown that peripheral membrane proteins can (transiently) form higher order structures due to membrane-mediated interactions. The clustering ability can be tuned via the penetration depth of the PMP's hydrophobic moiety and radius. To test our predictions experimentally, we propose the following approach: Using a set of well-characterized, fluorescently labeled PMPs that adsorb to the inner and/or outer leaflet of a liposome (e.g. a giant unilamellar vesicle, GUV), one may use fluorescence correlation spectroscopy (FCS) to quantify the degree of cluster formation. Using two PMP species with different fluorescent labels, say, a green and a red fluorophore, the formation of clusters can be detected on an almost single-molecule level by FCS via cross-correlating the red and green fluorescence signal [50]. Depending on the proximity of the fluorophores, also fluorescence resonance energy transfer (FRET) may be exploited to highlight clustering and even to determine the distance between PMPs. As both methods also work *in vivo*, assessing the clustering in living cells is also possible. Nevertheless, a cleaner and hence more quantitative setup with tunable properties of the bilayer, e.g. concerning lipid composition, is the GUV system.

Clearly, the above experimental approach requires well characterized PMPs. Here, several possibilities may be envisaged. Synthetic peptides, for which radius and hydrophobic length can be designed at will, or purified peripheral membrane proteins from cells are interesting candidates. Besides those proteins that have mentioned already in the Introduction, a particularly interesting probe may be the cholera toxin B subunit which binds 5 gangliosides and hence forms an effective PMP with a fairly large radius. Also, functionalized nanobeads with controlled surface properties may be used as PMP-like particles. Moreover, mutagenesis of particular PMPs in signaling pathways, e.g. reggies/flotilins, or the formation of chimera proteins may provide a means to probe our predictions *in vivo*.

## Materials and Methods

### Simulations

We have used a standard dissipative particle dynamics (DPD) model [51]–[53] to elucidate the behavior of peripheral membrane proteins. Two beads 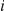 and 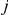 interacted via three pairwise linear forces 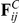, 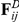, and 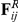 when their distance was 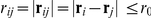. Beads with a distance 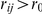 did not interact. The conservative repulsive force 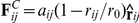 determined the degree of hydrophobicity of the beads. Here we have used interaction energies 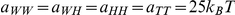 and 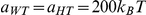 (indices indicate water bead (W), hydrophilic bead (H), and hydrophobic bead (T)) [53]. Here, 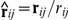 denotes the distance unit vector. Dissipative and random forces between two beads 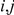 were described by 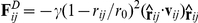 and 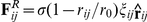. Here, 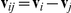 denotes the relative velocity of the two interacting beads, while 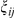 is an uncorrelated random variable with a zero mean and unit variance. Random and dissipative force act together as a termostat, with the friction coefficient and the amplitude of the noise being related by the fluctuation-dissipation theorem 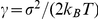. For our simulations we chose 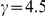, 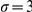.

Lipids were taken as linear polymers 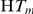 with a single hydrophilic head (H) and 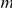 hydrophobic tail beads (T). Two succeeding beads 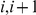 in 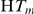 were connected by the attractive harmonic potential 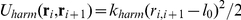, with the spring stiffness 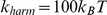 and the equilibrium bond length 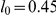. The rigidity of the polymer chain was obtained by a three-point bending potential 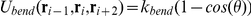, with the bond angle 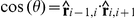 (bending constant 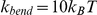). Peripheral membrane proteins were modeled as hexagonal cylinders consisting of a hydrophilic top layer and a hydrophobic moiety of 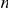 hydrophobic layers. All bead connections within the protein were taken as Hookean springs as described above. In our simulations we used protein lengths 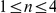. The diagonal of a protein's hexagonal cross section consisted of 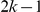 beads, i.e. protein radii were specified by the parameter 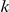.

The cutoff radius 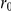, all bead masses and the thermostat temperature 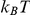 were set to unity. The bead density of the whole system was 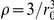, the initial lipid density in the membrane was chosen 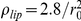. The linear size of the membrane patch was typically 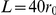, unless stated otherwise. In each run, we equilibrated the system for 50,000 time steps using a barostat to achieve a tensionless bilayer [54]. Then we fixed the equilibrated system size and monitored the system's behavior for another 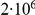 time steps. The equations of motion were integrated with a modified Velocity Verlet algorithm [52]. The time increment was 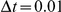. The conversion of the simulation units to SI units yielded 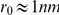 and 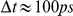; technical details may be found in [10]. In simulations with two lipid types 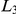 and 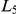, all repulsive forces between beads belonging to different lipid species were amplified by a factor 1.2 to induce domain formation of the longer lipid. Repulsive forces between lipid beads and protein or water beads remained unchanged.

The potential of mean force (PMF) of two PMPs in a membrane was calculated from the distribution 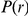 of inter-protein distances 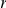 (within the plane of the membrane) via the relation 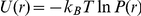. Umbrella sampling ensured a uniform sampling of the entire configurational space [40], [41]. During the umbrella sampling, harmonic potentials 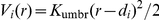 were imposed between the proteins. Here, 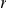 is the two-dimensional center-of-mass distance between the proteins in the plane of the membrane while 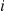 denotes the considered windows with center 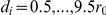. For each window, the system was equilibrated for 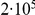 time steps and positional data were collected during the next 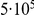 time steps. Subsequently, 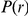 was determined by unbiasing and combining the distributions 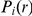 of each window using the weighted histogram analysis method (WHAM). A comprehensive description of the entire procedure can be found, for example, in Ref.[44]. The statistical error for the binding energy 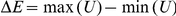 was less than 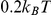 and hence significantly lower than thermal energy. Mean first passage times 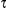 were determined from the PMF via
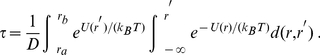
(2)Here, 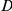 denotes the PMP's diffusion coefficient while 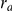 denotes the position of 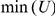; 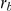 was the closest distance to 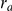 at which the PMF assumed the zero level (typically 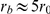).

## Supporting Information

Table S1Binding energy 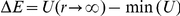 of PMPs residing in the same leaflet.(PDF)

Table S2Mean first passage time 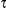 of PMPs residing in the same leaflet.(PDF)

Table S3Binding energy 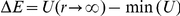 of PMPs residing in opposite leaflets.(PDF)

Table S4Mean first passage time 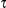 of PMPs residing in opposite leaflets.(PDF)
