# Supplementary material for: Dynamic Structure Formation of Peripheral Membrane Proteins
Source: PLoS Comput Biol. 2011 Jun 23;7(6):e1002067. doi: 10.1371/journal.pcbi.1002067 (PMC3121687; doi:10.1371/journal.pcbi.1002067)
Supplement: Table S1 — Binding energy of PMPs residing in the same leaflet. (PDF) [file pcbi.1002067.s001.pdf]

Hydrophobic length n	1	2	3	4
Radius $k = 2$	0.2	0.3	1.6	1.7
Radius $k = 3$	1.7	1.0	4.3	7.5
Radius $k = 4$	2.0	1.1	5.4	12.4
