# Supplementary material for: Dynamic Structure Formation of Peripheral Membrane Proteins
Source: PLoS Comput Biol. 2011 Jun 23;7(6):e1002067. doi: 10.1371/journal.pcbi.1002067 (PMC3121687; doi:10.1371/journal.pcbi.1002067)
Supplement: Table S2 — Mean first passage time of PMPs residing in the same leaflet. (PDF) [file pcbi.1002067.s002.pdf]

Hydrophobic length n	1	2	3	4
Radius $k = 2$	0.2	1.6	2.4	3
Radius $k = 3$	1.5	3.2	10	60
Radius $k = 4$	2	3.1	35	$6 \cdot 10^4$
