# Supplementary material for: Dynamic Structure Formation of Peripheral Membrane Proteins
Source: PLoS Comput Biol. 2011 Jun 23;7(6):e1002067. doi: 10.1371/journal.pcbi.1002067 (PMC3121687; doi:10.1371/journal.pcbi.1002067)
Supplement: Table S3 — Binding energy of PMPs residing in opposite leaflets. (PDF) [file pcbi.1002067.s003.pdf]

	Radius $k=2$				Radius $k=3$				Radius $k=4$			
4	4.0	0.0	0.0	0.0	17.6	1.2	1.7	2.2	33.0	6.3	4.0	8.5
3	1.9	0.3	0.0	0.0	9.8	3.3	1.5	1.7	22.1	10.2	2.4	4.0
2	0.0	0.2	0.3	0.0	2.3	4.0	3.3	1.2	8.2	12.3	10.2	6.3
1	0.0	0.0	1.9	4.0	0.7	2.3	9.8	17.6	1.2	8.2	22.1	33.0
n	1	2	3	4	1	2	3	4	1	2	3	4
