# Supplementary material for: Dynamic Structure Formation of Peripheral Membrane Proteins
Source: PLoS Comput Biol. 2011 Jun 23;7(6):e1002067. doi: 10.1371/journal.pcbi.1002067 (PMC3121687; doi:10.1371/journal.pcbi.1002067)
Supplement: Table S4 — Mean first passage time of PMPs residing in opposite leaflets. (PDF) [file pcbi.1002067.s004.pdf]

	Radius $k=2$				Radius $k=3$			
4	25	0	0	0	$2.4 \cdot 10^7$	2.2	1.3	1.2
3	3.6	0.2	0	0	$4.7 \cdot 10^4$	22	1.3	1.3
2	0	0.08	0.2	0	7.3	30	22	2.2
1	0	0	3.6	25	1.3	7.3	$4.7 \cdot 10^4$	$2.4 \cdot 10^7$
n	1	2	3	4	1	2	3	4

	Radius $k=4$			
4	$1.5 \cdot 10^{15}$	$1.1 \cdot 10^3$	6	260
3	$9 \cdot 10^9$	$1.1 \cdot 10^4$	3	6
2	$1.5 \cdot 10^4$	$4 \cdot 10^4$	$1.1 \cdot 10^4$	$1.1 \cdot 10^3$
1	80	$1.5 \cdot 10^4$	$9 \cdot 10^9$	$1.5 \cdot 10^{15}$
n	1	2	3	4
